# Effects of hypoxia-inducible factor-1α and matrix metalloproteinase-9 on alveolar-capillary barrier disruption and lung edema in rat models of severe acute pancreatitis-associated lung injury

**DOI:** 10.3892/etm.2014.1810

**Published:** 2014-06-26

**Authors:** BING QI, HAI-LONG CHEN, DONG SHANG, YING DONG, GUI-XIN ZHANG, LEI YU

**Affiliations:** 1 Dalian Medical University, Dalian, Liaoning 116044, P.R. China; 2 Department of Acute Abdominal Surgery, First Affiliated Hospital of Dalian Medical University, Dalian, Liaoning 116011, P.R. China; 3 Department of Gastroenterology and Hepatology, The Second Affiliated Hospital of Dalian Medical University, Dalian, Liaoning 116021, P.R. China

**Keywords:** acute pancreatitis, lung injury, hypoxia-inducible factor-1α, matrix metalloproteinase-9, alveolar-capillary barrier disruption, lung edema, inflammation

## Abstract

The aim of this study was to investigate the effects of hypoxia-inducible factor-1α (HIF-1α) and matrix metalloproteinase-9 (MMP-9) on alveolar-capillary barrier disruption and lung edema in rat models of severe acute pancreatitis-associated lung injury (PALI). A total of 40 male Sprague-Dawley rats were randomly divided into a sham surgery group (n=10) and three PALI groups, in which acute pancreatitis was induced by the retrograde infusion of 5% sodium taurocholate (1 ml/kg). The PALI groups were as follows: i) Untreated PALI group (n=10); ii) 2-methoxyestradiol (2ME2) group (5 mg/kg body mass; n=10); and iii) 2ME2 group (15 mg/kg body mass; n=10). In the two 2ME2 groups, the HIF-1α inhibitor 2ME2 was administered intraperitoneally 1 h after the induction of AP. The severity of the pancreatitis was evaluated by the serum amylase levels and pathology. The severity of the lung injury was evaluated by the wet/dry ratio, blood gas analysis and pathology. The alveolar-capillary barrier disruption was assessed by Evans blue dye extravasation. The protein and mRNA expression levels of HIF-1α and MMP-9 were studied using enzyme-linked immunosorbent assays (ELISAs), western blot analysis and reverse transcription-polymerase chain reaction. The active tumor necrosis factor-α levels were measured using an ELISA. The HIF-1α inhibitor 2ME2 attenuated the severity of the pancreatitis and PALI, while the lung edema and alveolar-capillary barrier disruption were significantly ameliorated compared with those in the untreated PALI group. Administration of the higher dose of 2ME2 significantly suppressed the protein expression of MMP-9 in the lung tissues. The results indicate that HIF-1α has a major function in alveolar-capillary barrier disruption and lung edema in PALI via a molecular pathway cascade involving MMP-9. Inhibition of HIF-1α by 2ME2 attenuates alveolar-capillary barrier disruption and lung edema. Pharmacological blockade of this pathway in patients with PALI may provide a novel therapeutic strategy.

## Introduction

Acute pancreatitis (AP) is an inflammatory disorder in which a complex cascade of immunological events develops, which affects the pathogenesis and clinical course of the disease, varying from a mild, self-limiting, transient illness to a severe, fatal outcome (1). Severe AP is characterized by pancreatic tissue necrosis, as well as complications including systemic inflammatory response syndrome and multiple organ dysfunction syndrome. Of the total number of patient mortalities due to severe AP, >50% are ascribed to acute lung injury in the early stage (2), also known as pancreatitis-associated lung injury (PALI).

Previous studies have demonstrated that pulmonary edema following PALI causes pulmonary swelling, which consequently produces secondary microvascular leakage, alveolar-capillary barrier disruption, and even alveolar damage and mortality (3–5). In severe PALI, pulmonary edema poses a critical clinical problem due to its association with acute respiratory function failure (6). Despite the fact that there have been significant advances in the understanding of the pathogenesis of alveolar-capillary barrier disruption and lung edema of PALI, as well as the availability of existing PALI treatments that attenuate the aforementioned derangements, the molecular mechanisms underlying this phenomenon remain poorly understood. The lack of effective drugs to ameliorate the initiation and progression of PALI-induced alveolar-capillary barrier disruption and lung edema has led to increased interest in the role that anti-edematous molecules may serve in alleviating this phenomenon (4,6–8).

Matrix metalloproteinases (MMPs) have an important function in the pathophysiology of alveolar-capillary barrier disruption and lung edema following PALI, and have become viable candidates for potential pharmacological targets (4,8,9). The expression of MMPs is crucial in numerous pathological events and has significant roles in a number of physiological processes, including remodeling of the extracellular matrix, degradation of type IV collagen in the basement membrane and migration of leukocytes during the immune and inflammatory responses (4,8,10,11).

Matrix metalloproteinase-9 (MMP-9), a subgroup of zinc-dependent endopeptidases, degrades components of the basement membrane, including collagen type IV, fibronectin and gelatin (12). Previous studies have demonstrated that the expression levels of MMP-9 in lung tissues of PALI model rats are markedly increased compared with those in healthy rat lungs (13). The proteolytic cleavage function of MMP-9 results in disruption of the alveolar-capillary barrier and lung edema (8). When the integrity of the alveolar-capillary barrier is compromised, vessel permeability increases and the alveolar-capillary barrier is no longer able to regulate the passage of molecules between the interstitial and lung parenchyma. This type of alveolar-capillary barrier dysregulation leads to an increase in the amount of water in the extracellular spaces of the lung tissue, namely, edema. In numerous types of lung pathologies, including PALI, the levels of MMP-9 are markedly upregulated. Thus, MMP-9 is considered to participate in the formation and progression of alveolar-capillary barrier disruption and lung edema (7,8). However, the molecular cascade leading to the upregulation of the levels of MMP-9 following the development of PALI has not been elucidated clearly.

Hypoxia-inducible factor 1 (HIF-1) is a highly conserved transcription factor that is present in almost all types of cell; it is strictly regulated by O_2_ availability. HIF-1 exists as a heterodimer consisting of hypoxia-inducible factor-1α (HIF-1α) and hypoxia-inducible factor-1β (HIF-1β) subunits. HIF-1β is ubiquitously expressed, whereas HIF-1α is observed at low levels under normoxic conditions (14). HIF-1α, an upstream transcription factor induced by hypoxia, regulates the subsequent expression of numerous types of proteins in response to the various pathophysiological conditions induced by hypoxia (4,15). Although previous studies have shown that the levels of HIF-1α are upregulated in AP (16,17), a study has also reported that HIF-1α is associated with augmented pulmonary vascular barrier disruption (18). However, the potential role of HIF-1α in lung tissue injury or restoration following the development of PALI remains unclear. Thus, whether HIF-1α contributes to the formation of alveolar-capillary disruption and lung edema by regulating the expression of MMP-9 in PALI remains to be clarified. The effects of HIF-1α following PALI also require further elucidation.

The purpose of the present study was to determine whether a molecular cascade involving HIF-1α and MMP-9 is causally associated with alveolar-capillary disruption and lung edema formation in a rodent model of PALI. This study further sought to provide novel data that may support potential therapeutic targets within this cascade for the alleviation of alveolar-capillary disruption and lung edema in PALI.

## Materials and methods

### Animals

Male Sprague-Dawley rats, 180–220 g in weight, were supplied by the Experimental Animal Center of Dalian Medical University (Dalian, China). The animals fasted overnight prior to the experiment, with water provided *ad libitum*. This study was conducted in strict accordance with the recommendations in the Guide for the Care and Use of Laboratory Animals of the National Institutes of Health (1st edition, published in 1996). The animal use protocol was reviewed and approved by the Institutional Animal Care and Use Committee of Dalian Medical University.

### Experimental design

A total of 40 male Sprague-Dawley rats were randomly divided into the sham surgery group (control group, n=10), in which the rats only underwent sham surgery, and three PALI groups (n=10 in each group), in which AP was induced by retrograde infusion of 5% sodium taurocholate (1 ml/kg). The PALI groups were as follows: i) Untreated PALI group; ii) animals treated with the HIF-1α inhibitor, 2-methoxyestradiol (2ME2; 5 mg/kg body mass; Selleck Chemicals, Houston, TX, USA); and iii) animals treated with 2ME2 (15 mg/kg body mass). 2ME2 was administered intraperitoneally 1 h after the induction of AP. All rats were sacrificed by femoral venous puncture 24 h after the induction of AP. Blood was immediately extracted from the abdominal aorta of the rats for blood gas analysis. Both lung tissues were also immediately collected and put into a freezing tube, which was then placed in liquid nitrogen and transferred to a refrigerator at −80°C. The frozen lung tissues were used for RT-PCR and Western blot evaluation.

### Induction of AP

AP was induced in 30 of the rats based on a previously described method (5), with minor modifications. Midline sterile laparotomy was performed under anesthesia with 10% chloral hydrate (Tianjin Kermel Chemical Reagent Co., Ltd., Tianjin, China) at a dose of 3 ml/kg by intraperitoneal injection. Sodium taurocholate (≤5%; 1 ml/kg; Sigma-Aldrich, St. Louis, MO, USA) was retrogradely infused into the distal end of the bile-pancreatic duct. The proximal bile duct was temporarily occluded at the hepatic portal by a vascular clamp for 5 min. Subsequently, the vascular clamp was removed and the duodenal and abdominal wounds were closed. The 10 rats in the sham surgery group only underwent a laparotomy.

### Serum amylase

In total, 10 rats were sacrificed to test the serum amylase. Serum was harvested from the collected blood by centrifugation using a high speed freezing centrifuge at 1,006 × g for 10 min. The serum was stored at −80°C prior to evaluation of the serum amylase levels using an automatic chemistry analyzer (Vitros 3600; Johnson & Johnson, Rochester, NY, USA).

### Wet/dry ratio

In total, 10 rats were sacrificed to test the wet/dry ratio. The magnitude of the pulmonary edema in the rats was determined by calculating the wet/dry ratio according to the following formula (7): Wet/dry ratio (%) = [(wet weight − dry weight)/dry weight] × 100, where the wet weight is the initial left lung weight and the dry weight is the weight of the lung following incubation at 72°C for 24 h.

### Evans blue dye extravasation

In total, 10 rats were sacrificed to test the Evans blue dye extravasation. The pulmonary microvascular permeability of the rats was measured using a modification of the Evans blue dye (Sigma-Aldrich) extravasation technique as previously described (19). Briefly, the rats were injected with 5% Evans blue dye through the internal jugular vein at a dose of 2 mg/100 g 30 min prior to sacrifice. Immediately after sacrifice, the lung tissues were collected and washed with normal saline. The lung tissues were then weighed after drying with filter paper. The Evans blue dye was extracted following homogenization in 1 ml deionized formamide and pulverization using an Ultrasonic Liquid Processor. A further 3 ml deionized formamide and 1 ml deionized formamide was added, and the mixture was incubated at 37°C for 48 h. The supernatant was separated by centrifugation at 1,000 × g for 5 min. The quantity of dye extracted was determined spectrophotometrically at 620 nm and calculated from a standard curve established with known amounts of Evans blue dye. Results are expressed as mg of dye per g of wet tissue.

### ELISA

A total of 10 rats was sacrificed to test the ELISA. The levels of HIF-1α, MMP-9 and tumor necrosis factor-α (TNF-α) in the serum were measured using commercially available ELISA kits (Shanghai Westang Bio-Tech Co., Ltd., Shanghai, China) according to the manufacturer’s instructions.

### Reverse transcription-polymerase chain reaction (RT-PCR)

Total RNA was extracted from the frozen tissue of the right lower lung lobe with chloroform and RNAiso Plus reagent [Takara Biotechnology (Dalian) Co., Ltd., Dalian, China] according to the manufacturer’s instructions. The lungs were extracted from 8 rats. The total RNA was solubilized in RNase-free water and quantified by NanoVue spectrophotometric measurement of the nucleic acids and proteins (GE Healthcare, Little Chalfont, UK). The purity of the RNA was assured by examining the optical density (OD)260/OD280 ratio. The RNA (2 μl) was reverse transcribed to complementary DNA using an RNA PCR kit (AMV) Ver. 3.0 [Takara Biotechnology (Dalian) Co., Ltd.] according to the manufacturer’s instructions. The PCR was performed using the primers presented in Table I. The amplification steps were as follows: Initial denaturation at 95°C for 3 min, 94°C denaturation for 30 sec, 51°C annealing for 30 sec and 72°C extension for 45 sec for 40 cycles, for HIF-1α; initial denaturation at 95°C for 3 min, 94°C denaturation for 30 sec, 60°C annealing for 30 sec and 72°C extension for 45 sec for 40 cycles, for MMP-9; and initial denaturation at 95°C for 3 min, 94°C denaturation for 30 sec, 52°C annealing for 30 sec and 72°C extension for 45 sec for 40 cycles, for β-actin. The PCR products (~5 μl) were electrophoresed using 1.7% agarose gel (Biowest, Barcelona, Spain) containing ethidium bromide (0.5 μg/ml; Biosharp Biotech Co., Hefei, China). The gels were visualized under UV light, and images of the gels were captured. The band intensities were determined by the ODs with individual PCR product/β-actin ratios.

### Western blot analysis

A total of 8 rats were used to extract the lungs. The frozen lung tissues were mechanically processed in lysis buffer (Nanjing KeyGen Biotech Co., Ltd., Nanjing, China) with protease inhibitors, phenylmethanesulfonyl fluoride and phosphatase inhibitor on ice using a protein extraction kit (Nanjing KeyGen Biotech Co., Ltd.) according to the manufacturer’s instructions. The total protein concentration was determined by NanoVue spectrophotometric measurement of the nucleic acids and proteins (GE Healthcare). Equal volumes (20 μl) of tissue extracts normalized by protein concentration were separated by electrophoresis through 10% sodium dodecyl sulfate-polyacrylamide gel (Bio-Rad Laboratories, Inc., Hercules, CA, USA) and transferred to polyvinylidene difluoride membranes. The membranes were blocked with 5% evaporated skimmed milk (BD Biosciences, Sparks, MD, USA) and then separately incubated with rabbit monoclonal antibody against HIF-1α (1:1,000; Epitomics Inc., Burlingame, CA, USA) and rabbit monoclonal antibody against MMP-9 (1:1,000; Epitomics Inc.). Equal loading of protein was confirmed and adjusted by β-actin monoclonal antibody (1:1,000; Wuhan Boster Biological Technology, Ltd., Wuhan, China). These antibodies were incubated with the membrane at room temperature for 2 h. Following three wash cycles in Tris-buffered saline with Tween 20, the membrane was incubated with secondary antibody peroxidase-conjugated AffiniPure goat anti-rabbit IgG (1:5,000; ZSGB-BIO, Beijing, China). To quantify the relative expression levels of the target protein, blot images were captured and analyzed using an image analysis program (BioSpectrum AC 410; UVP, LCC, Upland, CA, USA). The expression intensities of the proteins from the different groups were statistically compared.

### Histological examination

In total 8 rats were used to extract the lungs. Morphological alterations in the lungs and pancreas were examined in individual rats from each of the four groups. The lungs and pancreas were fixed with 10% formalin and embedded in paraffin. Paraffin sections (4 μm thick) were stained with hematoxylin and eosin (H&E) for examination by light microscopy (model TS100, Nikon, Japan). A scoring system to grade the degree of lung and pancreatic injury was employed (20,21). In this system, the lung injury was assessed from the degree of edema, inflammatory cell infiltration and bleeding, each of which was scored on a scale of 0–3, and the pancreatic injury was assessed from the degree of edema, inflammation, vacuolization and necrosis, each of which was scored on a scale of 0–4. The grading was performed by a blinded pathologist. The injury scores were calculated by adding the individual scores for each category.

### Statistical analysis

All data are presented as the mean ± standard deviation. Statistical analysis was performed with SPSS software for Windows, version 16.0 (SPSS, Inc., Chicago, IL, USA). The differences among multiple groups were assessed using χ^2^ analysis with P<0.05 considered to indicate a statistically significant difference.

## Results

### General information

Compared with those of the control group, the levels of serum amylase, PaCO_2_ and the wet/dry ratio increased and PaO_2_ decreased in the untreated PALI group. Following the administration of the HIF-1α inhibitor, the levels of serum amylase, PaCO_2_ and the wet/dry ratio decreased, whereas the PaO_2_ increased, compared with those in the untreated PALI group. Furthermore, the effect of the larger dose of the HIF-1α inhibitor was stronger than that of the smaller dose (Table II).

### Lung permeability

**Figure 1:**
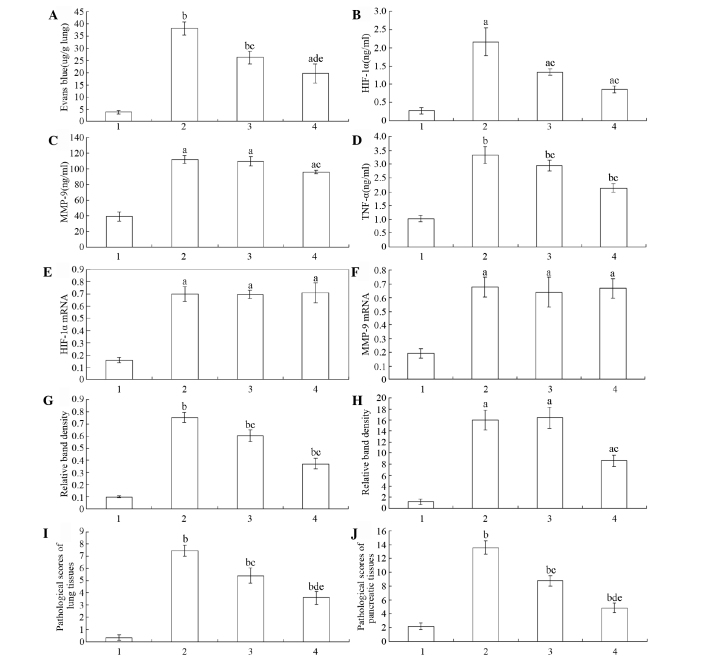
Levels of Evans blue dye extravasation, active HIF-1α, MMP-9, TNF-α expression, HIF-1α and MMP-9 mRNA and protein expression, and pathological scores of lung and pancreatic tissues in the various groups. (A) Capillary permeability measured by Evans blue dye extravasation in the various groups. Serum expression levels of (B) active HIF-1α, (C) MMP-9 and (D) TNF-α in the various groups. Expression levels of (E) HIF-1α and (F) MMP-9 mRNA in lung tissues and of (G) HIF-1α and (H) MMP-9 protein in lung tissues. Pathological scores of the (I) lung and (J) pancreatic tissues in the various groups. Data are presented as the mean ± standard deviation (n=10). 1, control group; 2, untreated PALI group; 3, PALI + 2ME2 (5 mg/kg); 4, PALI + 2ME2 (15 mg/kg). ^a^P<0.05, ^b^P<0.01 vs. the sham surgery group; ^c^P<0.05, ^d^P<0.01 vs. the PALI group. HIF-1α, hypoxia inducible factor-1α; MMP-9, matrix metalloproteinase-9; TNF-α, tumor necrosis factor-α; PALI, pancreatitis-associated lung injury; 2ME2, 2-methoxyestradiol.

The rats in the untreated PALI group showed a significant increase in the microvascular capillary permeability of the lung compared with that of the sham surgery group. Following the administration of the HIF-1α inhibitor 2ME2 (5 mg/kg), the capillary leakage was reduced significantly compared with that of the untreated PALI group. Compared with that of the PALI + 2ME2 (5 mg/kg) group, the blue dye accumulation in the lung parenchyma of the PALI + 2ME2 (15 mg/kg) group was significantly reduced (Fig. 1A).

### Levels of active HIF-1α, MMP-9 and TNF-α

For the quantitative determination of the active levels of HIF-1α, MMP-9 and TNF-α in the serum, a fluorescence ELISA designed to measure the HIF-1α, MMP-9 and TNF-α activity levels was used. The results showed that compared with those of the control group, the levels of HIF-1α, MMP-9 and TNF-α increased in the untreated PALI group. Following the administration of the HIF-1α inhibitor, the levels of HIF-1α, MMP-9 and TNF-α decreased compared with those of the untreated PALI group. Furthermore, the effect of the larger dose of the HIF-1α inhibitor was stronger than that of the smaller dose (Fig. 1B–D).

### HIF-1α and MMP-9 mRNA expression levels

**Figure 2:**
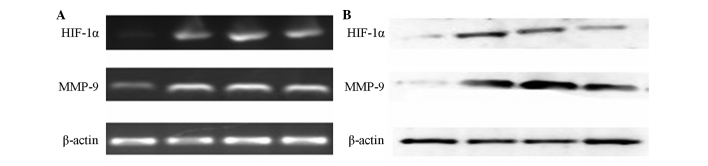
Expression levels of HIF-1α and MMP-9 mRNA, and HIF-1α and MMP-9 protein in the lung tissues of the rats in the various groups. (A) Expression levels of HIF-1α and MMP-9 mRNA. (B) Expression levels of HIF-1α and MMP-9 protein. HIF-1α, hypoxia inducible factor-1α; MMP-9, matrix metalloproteinase-9.

The RT-PCR showed that the mRNA expression levels of HIF-1α and MMP-9 were upregulated significantly in the untreated PALI group compared with those in the control group. However, the HIF-1α and MMP-9 mRNA levels in the lung tissues did not exhibit significant differences between the untreated PALI group and the two 2ME2 groups (Figs. 1E, 1F and 2A).

### HIF-1α and MMP-9 protein expression levels

HIF-1α expression levels are reportedly upregulated during AP (16,17). HIF-1α was almost undetectable in the lung tissues of the rats in the sham surgery group. The HIF-1α and MMP-9 protein expression levels showed significant increases in the untreated PALI group compared with those in the control group. Compared with those in the untreated PALI group, the HIF-1α protein expression levels were significantly reduced in the PALI + 2ME2 (5 mg/kg) and PALI + 2ME2 (15 mg/kg) groups. However, the difference in the MMP-9 protein levels between the untreated PALI and PALI + 2ME2 (5 mg/kg) groups was weakly distinguishable, whereas in the PALI + 2ME2 (15 mg/kg) group, the MMP-9 protein expression levels were significantly reduced compared with those in the untreated PALI group (Figs. 1G, 1H and 2B).

### Histological examination

The lung and pancreatic tissues were sectioned, stained with H&E and scored for edema, acinar necrosis, hemorrhage and fat necrosis, inflammation and perivascular infiltration (Table III).

**Figure 3:**
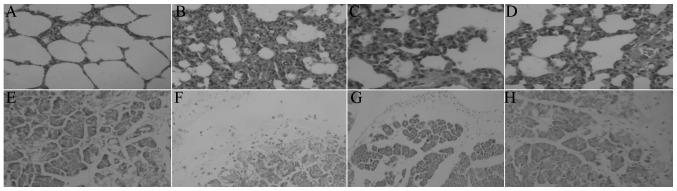
Representative photomicrographs of the lungs and pancreas in the various groups. The pathology of the lung (A–D) and pancreatic (E–H) tissues in the (A,E) control group, (B, F) untreated PALI group, (C, G) PALI + 2ME2 (5 mg/kg) group, and (D, H) PALI + 2ME2 (15 mg/kg) group. H&E staining; magnification, ×20. PALI, pancreatitis-associated lung injury; 2ME2, 2-methoxyestradiol, H&E, hematoxylin and eosin.

The rats in the untreated PALI group showed significant increases in pathological scores of lung and pancreatic tissues compared with those in the sham surgery group. Compared with those in the PALI + 2ME2 (5 mg/kg) group, the pathological scores of the lung and pancreatic tissues in the PALI + 2ME2 (15 mg/kg) group were significantly reduced (Figs. 1I and 3A–D, and Figs. 1J and 3E–H for lung and pancreas, respectively).

## Discussion

Previous studies have demonstrated that HIF-1α may regulate MMP-9 expression in the pathogenesis of blood-brain barrier disruption and brain edema following brain injury (4,22). Bai *et al* (16) observed that HIF-1α has an important function in the pathogenesis of AP. Keck *et al* (13) found that MMP-9 also participates in the pathogenesis of PALI. Thus, whether HIF-1α contributes to the formation of lung edema by regulating MMP-9 requires clarification.

The major finding of the present study is that the concurrently elevated expression levels of HIF-1α and MMP-9 in the lung tissues of rat models of PALI temporally coincided with lung edema formation and alveolar-barrier disruption. In the PALI model used in the present study, the edema, determined by the wet/dry ratio, was reduced by the HIF-1α-selective inhibitor, 2ME2, compared with that in the untreated PALI group. In addition, the alveolar-barrier disruption determined by Evans blue dye extravasation was also ameliorated by the inhibition of HIF-1α by 2ME2. At the same time, the inhibition of HIF-1α caused reduced levels of serum amylase, PaCO_2_ and pathological scores of the tissues of the lung and pancreas, as well as increased PaO_2_ compared with those in the untreated PALI group.

The data in the present study further support the hypothesis that HIF-1α affects the pathogenesis of PALI, which is consistent with the findings of previous studies (14,16,18). Blocking HIF-1α expression may prevent the formation of lung edema and alveolar-capillary barrier disruption, alleviate PALI and provide a novel approach for the treatment of AP.

Furthermore, the larger dose of the HIF-1α inhibitor downregulated the protein expression levels of MMP-9 in the PALI model compared with those in the untreated PALI group. The findings of the present study suggest a functional interaction linking MMP-9 and HIF-1α, which is possibly dysregulated in PALI. The data also suggest that HIF-1α regulates the expression levels of MMP-9, which are crucial in edema formation and alveolar-capillary barrier disruption in PALI.

The large quantities of inflammatory factors that enter the lung tissues in PALI cause damage and necrosis of lung epithelial cells, microvascular endothelial cells and tight junctions. Furthermore, pancreatic proteinase activates the complementary systems, indirectly leading to increased tissue damage effects and pulmonary transudate, which cause a reduction in the pulmonary functions of ventilation. Thus, hypoxia may be a severe consequence of PALI, leading to aberrations in lung function and repair (15). Hypoxia also results in damage of the alveolar lining layer, apoptosis of alveolar epithelial cells, lung edema, increased vascular permeability and augmented barrier disruption (14,18).

**Figure 4:**
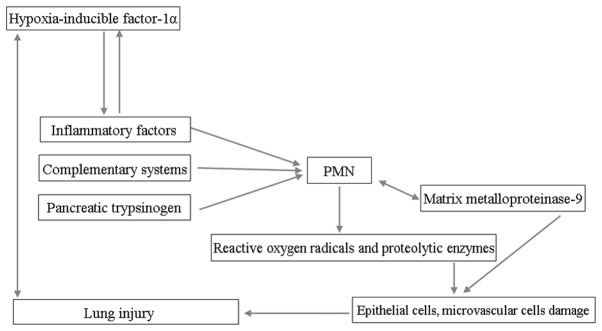
Mechanisms of HIF-1α that contribute to the upregulation of the MMP-9 expression levels, alveolar-capillary barrier disruption and lung edema. PMN, polymorphonuclear leukocytes; HIF-1α, hypoxia inducible factor-1α; MMP-9, matrix metalloproteinase-9.

Previous studies have demonstrated that hypoxia elicits tissue inflammation (23,24). For example, Hartmann *et al* (24) demonstrated that exposure to a high altitude is associated with elevated levels of inflammatory mediators in humans. Similarly, mice that are exposed to acute hypoxia (e.g., 8% oxygen over 8 h) develop elevated plasma levels of cytokines, in conjunction with pulmonary edema and inflammatory cell accumulation in the lungs and other organs (9,25). In the inflammatory response, a large dose of cytokines (TNF-α, IL-1β and IL-8) and serine proteases derived from the pancreas may activate polymorphonuclear leukocytes (PMNs), which secrete significant amounts of MMP-9. Subsequently, MMP-9 promotes PMN migration, alveolar capillary leakage and lung edema (13,26) (Fig. 4).

2ME2 is a naturally endogenous-occurring metabolite of estradiol, which post-transcriptionally downregulates the expression levels of HIF-1α and is an anti-angiogenic and antitumor agent (27,28). Studies have provided *in vitro* and *in vivo* evidence that 2ME2 has a direct effect on the inhibition of HIF-1α and the inhibition is not the result of a ‘side-effect’ of mitotic arrest (29,30). Furthermore, 2ME2 treatment reduces the levels of nuclear and total HIF-1α proteins in a dose-dependent manner. The downregulated expression levels of HIF-1α post-transcription may be the reason for the absence of statistical significance between the HIF-1 and MMP-9 mRNA expression levels following the administration of 2ME2 and in the untreated PALI group in the present study. 2ME2 inhibits HIF-1α translation and nuclear translocation, thereby suppressing the inflammatory response, PMN activation and MMP-9 expression (27).

The aforementioned results suggest that HIF-1α may be an upstream protein that causes the alveolar-capillary barrier disruption and lung edema formation in PALI through its regulatory expression of catalytic enzymes, including MMP-9.

In conclusion, the present study demonstrated in a PALI model that an HIF-1α-MMP-9 signaling cascade exists, wherein PALI first triggers induction of HIF-1α, which in turn upregulates MMP-9 expression levels and leads to pathophysiological alveolar-capillary barrier disruption and lung edema. Inhibition of HIF-1α may provide novel insights into PALI. These findings enhance our knowledge of the prevention of acute lung injury secondary to severe AP.

## Figures and Tables

**Table I:** Sequences of the primers.

Cytokines	Product size (bp)	Sense primer	Antisense primer
HIF-1α	670	5′-GGCAACGAGAAGAAAAATAGG-3′	5′-GAGGAATGGGTTCACAAATC-3′
MMP-9	196	5′-CCCTGCGTATTTCCATTCATC-3′	5′-ACCCCACTTCTTGTCAGCGTC-3′
β-actin	201	5′-CGTTGACATCCGTAAAGAC-3′	5′-TGGAAGGTGGACAGTGAG-3′

HIF-1α, hypoxia-inducible factor-1α; MMP-9, matrix metalloproteinase-9.

**Table II:** Comparison of serum amylase levels, blood gas analysis and wet/dry weight ratio of lung tissue in the various groups.

Groups	PaO_2_/mmHg	PaCO_2_/mmHg	AMY (U/l)	W/D
Control	98.05±1.13	24.99±2.08	1864.5±385.22	0.87±0.31
PALI	74.72±2.30^a^	50.34±2.31^a^	9428.1±302.80^a^	2.86±0.43^a^
PALI + 2ME2 (5 mg/kg)	83.01±2.83^a^,^b^	40.16±1.10^a^,^b^	7048.1±847.51^a^,^b^	2.45±0.35^a^,^c^
PALI + 2ME2 (15 mg/kg)	89.12±2.44^a^,^c^	32.01±1.24^a^,^b^	3923.8±191.12^a^,^c^	1.94±0.28^a^,^c^

a P<0.01 vs. the sham surgery (control) group;

b P<0.05,

c P<0.01 vs. the PALI group. 1 mmHg = 0.133 kPa. Data are presented as the mean ±SD.

AMY, amylase; W/D, wet/dry ratio; PALI, pancreatitis-associated lung injury; 2ME2, 2-methoxyestradiol.

**Table III:** Relative levels of the pathological scores of the lung and pancreatic tissues of the rats in the various groups.

Groups	Lung tissues	Pancreatic tissues
Control	0.3±0.23	2.15±0.47
PALI	7.5±0.44^a^	13.55±1.01^a^
PALI + 2ME2 (5 mg/kg)	5.4±0.61^a^,^b^	8.75±0.75^a^,^b^
PALI + 2ME2 (15 mg/kg)	3.6±0.52^a^,^c^,^d^	4.85±0.71^a^,^c^,^d^

a P<0.01 vs. the sham surgery (control) group;

b P<0.05,

c P<0.01 vs. the PALI group;

d P<0.01 vs. the PALI + 2ME2 (5 mg/kg) group.

PALI, pancreatitis-associated lung injury; 2ME2, 2-methoxyestradiol.
